# Temporal dynamics of gene expression during the development of Campylobacter jejuni biofilms

**DOI:** 10.1099/mgen.0.001387

**Published:** 2025-05-06

**Authors:** Manca Volk, Ozan Gundogdu, Anja Klančnik

**Affiliations:** 1 Department of Food Science and Technology, Biotechnical Faculty, University of Ljubljana, Ljubljana, Slovenia; 2 Faculty of Infectious and Tropical Diseases, London School of Hygiene & Tropical Medicine, London, UK

**Keywords:** biofilm development, biofilms, *Campylobacter jejuni*, gene expression, microbial regulation, temporal dynamics, transcriptomics

## Abstract

*Campylobacter jejuni,* an important pathogen of bacterial gastrointestinal infections, forms biofilms that enable its survival in different environments. *C. jejuni* biofilm development is still poorly understood, and thus, in this study, we characterized gene expression changes at different biofilm stages using RNA sequencing. Early biofilms (after 16 and 24 h) showed increased expressions of genes involved in cysteine and methionine metabolism, whereas mature biofilms (after 48 and 72 h) showed decreased expression of genes encoding capsular polysaccharides and lipooligosaccharides. Both early and mature biofilms showed increased expressions of genes involved in flagella formation, leucine metabolism and the oxidative stress response and decreased expressions of genes involved in energy metabolism, iron acquisition and transmembrane transport. This study provides insights into the molecular mechanisms underlying *C. jejuni* biofilm maturation, environmental resistance and the dynamic nature of gene expression during biofilm development.

**Impact Statement**

*Campylobacter jejuni* is a leading cause of bacterial gastroenteritis worldwide, with its ability to form biofilms contributing significantly to its survival and persistence in diverse environments. Despite its importance, the molecular mechanisms underlying biofilm development remain poorly understood. This study leverages transcriptomic analysis to investigate *C. jejuni* biofilm development over time, offering novel insights into the molecular mechanisms underlying biofilm development. By revealing stage-specific adaptations, our findings expand the understanding of how *C. jejuni* transitions through early to mature biofilm stages to enhance its persistence and resistance in diverse environments. These findings provide a foundation for developing targeted strategies to disrupt key processes identified in this work, mitigating biofilm development and reducing biofilm-associated contamination in food processing environments, thereby improving food safety.

## Data Summary

Sequencing data/differential gene expression were deposited in the Gene Expression Omnibus database (accession number GSE272440, available at https://www.ncbi.nlm.nih.gov/geo/). The code for alignment and differential expression analysis is available at https://github.com/NIB-SI/CampyDEA.

## Introduction

*Campylobacter jejuni* is a Gram-negative microaerophilic bacterium that causes campylobacteriosis, which is the most common bacterial gastrointestinal infection and cause of foodborne zoonoses worldwide [1,2]. *C. jejuni* inhabits the gastrointestinal tract of both avians and mammals. Human infections in high-income countries are predominantly linked to the handling and consumption of poultry products. Raw milk and contaminated water are also important sources, particularly for low- and middle-income countries [3]. One of the proposed mechanisms that enable *C. jejuni* to persist in the environment is its ability to form biofilms, which enhances its stress tolerance and antimicrobial resistance [4]. These biofilms can form on various surfaces, especially those used in the food processing industry, such as stainless steel, glass and polystyrene [1]. Despite the significant role of biofilms in the survival and persistence of *C. jejuni* in the environment, the mechanisms underlying biofilm development remain unclear.

In biofilms, cells are surrounded by an extracellular polymeric matrix that acts as a physical barrier and provides protection against harsh environmental conditions [1]. The extracellular polymeric matrix of *C. jejuni* biofilms, similar to those of other bacterial biofilms, consists of proteins, carbohydrates and environmental DNA (eDNA) [5,6]. *C. jejuni* forms strain-specific mono-species biofilms that can exist as pellicles on liquid surfaces, as aggregates in liquids, or attached to surfaces in liquid cultures, thereby increasing survival compared to their planktonic counterparts [7]. Furthermore, *C. jejuni* can also form multispecies biofilms [8,9]. Biofilm formation is affected by various factors, including nutrient availability, osmolarity, temperature and oxygen tension [10,12]. The ability to form biofilms and their ultrastructure are strain-dependent. After 24 h under microaerobic conditions, the *C. jejuni* NCTC11168 strain formed a multilayered biofilm structure, whereas the 81–176 strain formed finger-like biofilm structures. After 48 h, the biofilms increased in height and biomass in both strains [12]. The onset of biofilm formation is characterized by the appearance of eDNA, which contributes to the structural development of biofilms. eDNA also plays a crucial role in biofilm maturation and can facilitate horizontal gene transfer, as increased DNA exchange has been observed in biofilms. However, it is still unclear whether DNA uptake and recombination processes are upregulated in biofilms [13].

The molecular mechanisms that regulate *C. jejuni* biofilm formation and maturation are not yet fully understood [5,12, 13]. Nevertheless, the genes reported to be involved in biofilm formation and maturation are related to cell motility (*flaA*, *flaB*, *flaC*, *flaG*, *fliA*, *fliS* and flhA), cell surface modifications (*peb4*, *pgp1* and *waaF*), quorum sensing (*luxS*) and stress responses (*ppk1*, *spoT*, *cj1556*, *csrA*, *cosR* and *cprS*) [12]. Proteins involved in the motility complex, including flagellins (FlaA and FlaB), the filament cap (FliD), basal body (FlgG and FlgG2) and chemotactic protein (CheA), showed higher expression in *C. jejuni* biofilms compared to planktonic cells in the stationary phase [14]. Other proteins with increased expression in biofilms are involved in the general stress response (GroEL and GroES), oxidative stress response (Tpx and Ahp), adhesion (Peb1 and FlaC), biosynthesis (PurL, NifU, EF-G, riboflavin synthase and ribosomal release factor), energy production and catabolic processes [14]. Transcriptomic and proteomic analysis of *C. jejuni* biofilms grown under aerobic conditions, compared to planktonic cells grown under microaerobic conditions, revealed altered expression of genes and proteins involved in iron acquisition and metabolism, glycan production and attachment, energy metabolism, aa catabolism and chemotaxis [15].

Although the spatial and temporal organization of *C. jejuni* biofilms is known, the temporal dimension of gene expression underlying biofilm formation and maturation is currently unclear. Previous studies involving transcriptomic and proteomic analyses at different stages of *Bacillus subtilis* and *Pseudomonas putida* biofilms have shown dynamic shifts in gene expression over time [16]. The above-mentioned specific physiological responses and molecular mechanisms of *C. jejuni* enable its unique adaptive strategies during biofilm development. Thus, it is crucial to comprehensively understand the dynamics of gene expression during biofilm development over time. In the present study, we used RNA sequencing (RNA-seq) to investigate the changes in gene expression at different stages of biofilm development (16, 24, 48 and 72 h), focusing on functional analysis to determine the gene functions present at each stage. We hypothesize that biofilm development involves distinct stage-specific transcriptional adaptations that promote environmental persistence and stress tolerance. By identifying these molecular changes, we aim to provide a foundation for understanding the regulatory networks driving biofilm formation and maturation in *C. jejuni*.

## Results

### RNA-seq overview

**Fig. 1.**
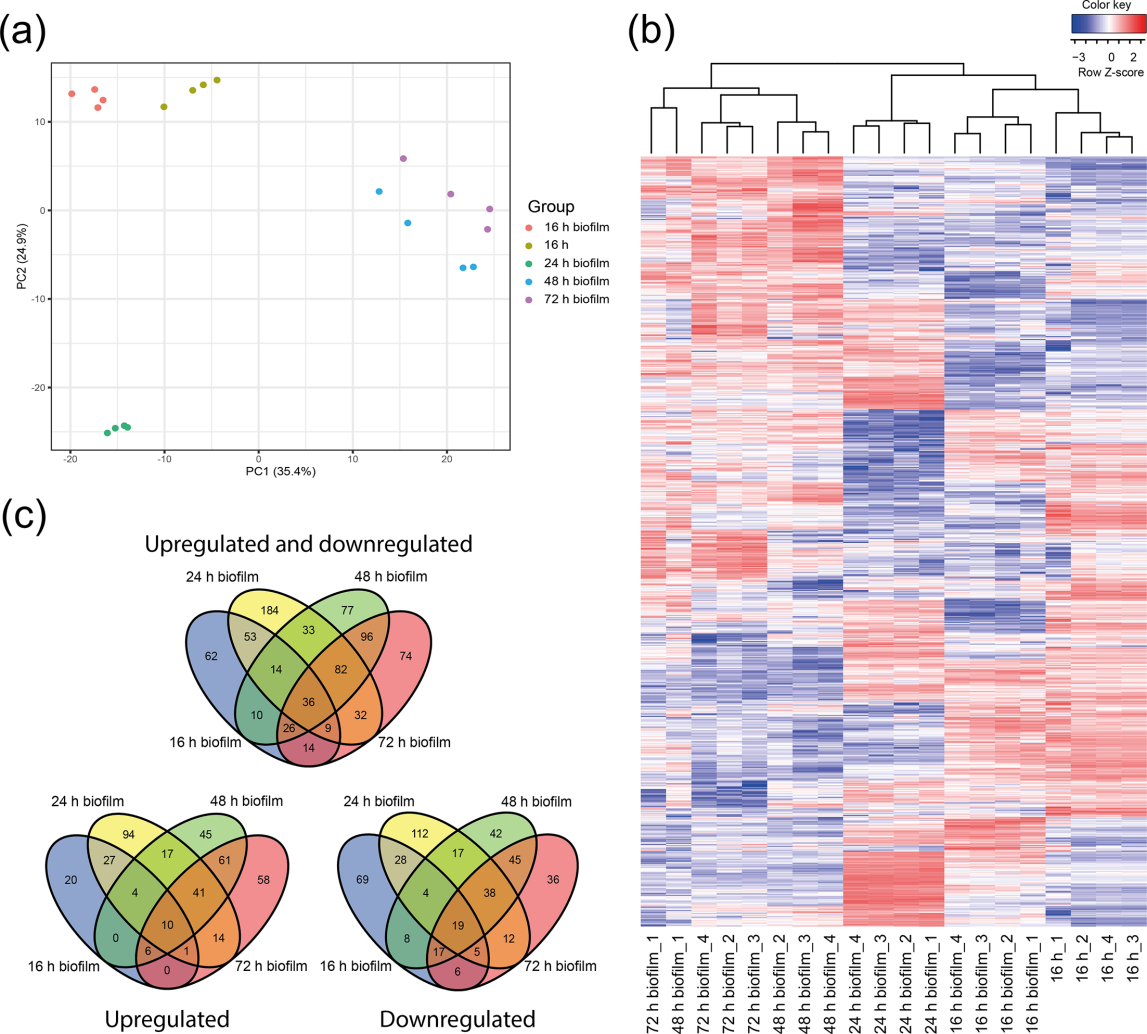
Detailed analyses of DEGs. (a) PCA showing five distinct transcriptional profiles: 16 h planktonic cells (olive), 16 h biofilm (red), 24 h biofilm (green), 48 h biofilm (blue) and 72 h biofilm (purple). (b) A heatmap of relative expression of all DEGs in all biological replicates. Log_2_ c.p.m. is centred and scaled by row. Genes with relatively high and low expression are highlighted in red and blue, respectively. (c) Venn diagrams depicting the overlap of upregulated and downregulated genes (top), upregulated genes (bottom left) and downregulated genes (bottom right) at all time points compared to the planktonic culture.

We generated more than 13.3 million paired-end 150 bp Illumina reads per sample from RNA extracted from 16, 24, 48 and 72 h biofilms, totalling 337.5 million reads with Phred quality scores greater than Q30 for over 91% of the bases. In 19 samples, 70.4–91.3% of the reads aligned to the reference sequence, whereas one sample (24 h biofilm_2) contained only 37.2% aligned reads (Table S1, available in the online Supplementary Material). Despite the lower number of aligned reads, the sample was included in the sequential analysis after confirming its biological consistency with other replicates through principal component analysis (PCA) and hierarchical clustering. The inclusion of this sample did not affect the clustering patterns or the reliability of downstream results. PCA (Fig. 1a) showed low variance between biological replicates and revealed four clusters corresponding to planktonic cells (16 h), 16 h biofilm, 24 h biofilm and 48/72 h biofilm. To visualize the changes in expression across all samples, a heatmap with hierarchical clustering was generated (Fig. 1b).

Of the 1504 genes transcribed under all conditions, 224 (14.9%) were differentially expressed in the 16 h biofilm, 443 (29.5%) in the 24 h biofilm, 374 (24.9%) in the 48 h biofilm and 369 (24.5%) in the 72 h biofilm. In the 16 h biofilm, 68 (30.4 %) genes were upregulated, and 156 (69.6%) genes were downregulated. In the 24 h biofilm, 208 (47.0 %) genes were upregulated, and 235 (53.0 %) genes were downregulated. In the 48 h biofilm, 184 (49.2 %) genes were upregulated, and 190 (50.8%) were downregulated. In the 72 h biofilm, 191 (51.8 %) genes were upregulated, and 178 (48.2%) were downregulated. The complete RNA-seq dataset is available in Data S1.

Across all time points, 36 common differentially expressed genes (DEGs) were identified: 10 were upregulated, and 19 were downregulated. Across time points 24, 48 and 72 h, 82 common DEGs were identified, 41 were upregulated and 38 were downregulated. At the 48 h and 72 h time points, 96 common DEGs were identified, 61 were upregulated and 45 were downregulated. The numbers for common DEGs, upregulated DEGs and downregulated DEGs reflect different subsets and overlapping groups across various time points (Fig. 1c). A list of all common DEGs can be found in Data S2.

### Top 10 DEGs

The top 10 DEGs are listed in Table 1.

**Table 1.** The top 10 most DEGs and their functions in 16, 24, 48 and 72 h biofilms. Log_2_ FC, log_2_-fold change; CPS, capsular polysaccharide; LOS, lipooligosaccharide

Biofilm	Function	Locus tag	Gene name	Log_2_ FC	Biofilm	Function	Locus tag	Gene name	Log_2_ FC
**16 h biofilm**	aa transport	*Cj0025c*		+3.98	**24 h biofilm**	Electron transport chain	*Cj0037c*		+4.98
	Transport	*Cj0263*	*zupT*	+2.47		aa transport	*Cj0025c*		+4.71
	Unknown	*Cj1406c*		+2.45		aa metabolism	*Cj1718c*	*leuB*	+4.11
	aa metabolism	*Cj1726c*	*metAA*	+2.37		aa metabolism	*Cj1717c*	*leuC*	+4.03
	Lipid metabolism	*Cj0915*		+2.36		Unknown	*Cj0737*	*p95*	+4.03
	aa metabolism	*Cj1201*	*metE*	+2.36		Fe-S cluster biosynthesis pathway	*Cj0240c*	*iscS*	+3.91
	Fe-S cluster biosynthesis pathway	*Cj0239c*	*nifU*	+2.21		Oxidative stress response	*Cj0241c*	*herA*	+3.69
	Transport	*Cj0982c*	*cjaA*	+2.14		Signal transduction	*Cj1170c*	*omp50*	+3.68
	aa metabolism	*Cj0912c*	*cysM*	+2.06		Unknown	*Cj1406c*		+3.65
	Nitrosative stress response	*Cj1586*	*cgb*	+2.01		Oxidative stress response	*Cj1385*	*katA*	+3.62
	Iron transport	*Cj1614*	*chuA*	−3.25		Energy metabolism	*Cj0439*	*mfrE*	−2.67
	Virulence	*Cj1450*	*ciaI*	−3.26		Transport	*Cj0045c*		−2.68
	Heat shock response	*Cj0758*	*grpE*	−3.34		Phosphate transport	*Cj0614*	*pstC*	−2.80
	Energy metabolism	*Cj0439*	*mfrE*	−3.38		Energy metabolism	*Cj0438*	*mfrB*	−2.90
	Energy metabolism	*Cj0438*	*mfrB*	−3.58		Unknown	*Cj1384c*		−3.12
	Nitrogen metabolism	*Cj1358c*	*nrfH*	−3.83		nt transport and metabolism	*Cj0594c*		−3.15
	Energy metabolism	*Cj0437*	*mfrA*	−3.92		Unknown	*Cj1493c*		−3.19
	Heat shock response	*Cj0757*	*hrcA*	−4.57		C4-dicarboxylate transport	*Cj0671*	*dcuB*	−3.90
	Heat shock response	*Cj0509c*	*clpB*	−4.58		Energy metabolism	*Cj0437*	*mfrA*	−4.09
	Unknown	*Cj0735*		−4.64		Unknown	*Cj0735*		−6.06
**48 h biofilm**	Defence mechanism	*Cj0424*		+4.60	**72 h biofilm**	Protein translocation	*Cj0472*	*secE*	+4.23
	Unknown	*Cj0040*		+4.38		Virulence	*Cj1242*	*ciaC*	+3.55
	Cell motility	*Cj0041*	*fliK*	+3.83		Translation	*Cj0155c*	*rpmE*	+3.32
	Virulence	*Cj1242*	*ciaC*	+3.80		Cell motility	*Cj0041*	*fliK*	+3.19
	Cell motility	*Cj0528c*	*flgB*	+3.62		aa metabolism	*Cj1718c*	*leuB*	+3.16
	aa metabolism	*Cj1201*	*metE*	+3.25		Defence mechanism	*Cj0424*		+3.14
	Cell motility	*Cj1729c*	*flgE2*	+3.25		Bacterial secretion system	*Cj0683*		+3.12
	Translation	*Cj1711c*	*rsmA*	+3.17		Transport	*Cj1200*		+3.07
	Cell motility	*Cj0697*	*flgG2*	+3.10		aa metabolism	*Cj1717c*	*leuC*	+3.07
	Transport	*Cj1200*		+3.09		Unknown	*Cj0331c*		+2.86
	Phosphate transport	*Cj0615*	*pstA*	−2.59		aa metabolism	*Cj1437c*		−3.58
	Iron transport	*Cj0175c*	*cfbpA*	−2.60		Energy metabolism	*Cj1585c*		−3.64
	Cell motility	*Cj1675*	*fliQ*	−2.74		LOS outer core biosynthesis	*Cj1142*	*neuC1*	−3.83
	Iron transport	*Cj1630*	*tonB2*	−2.98		LOS outer core biosynthesis	*Cj1144c*		−3.94
	Iron transport	*Cj1617*	*chuD*	−2.98		Flagellar glycosylation	*Cj1324*		−4.13
	Unknown	*Cj1384c*		−3.08		Phosphate transport	*Cj0614*	*pstC*	−4.23
	nt transport and metabolism	*Cj0513*	*purS*	−3.49		CPS biosynthesis	*Cj1439c*	*glf*	−4.43
	Phosphate transport	*Cj0614*	*pstC*	−3.51		CPS biosynthesis	*Cj1422c*		−4.53
	Unknown	*Cj0818*		−3.54		LOS outer core biosynthesis	*Cj1136*		−4.86
	Phosphate transport	*Cj0613*	*pstS*	−4.49		Phosphate transport	*Cj0613*	*pstS*	−5.12

Gene functions were assigned according to the Kyoto Encyclopedia of Genes and Genomes (KEGG), Gene Ontology (GO), Clusters of Orthologous Genes or the literature. In 16 h biofilms, upregulation was observed for genes involved in aa transport (*Cj0025c*), aa metabolism (*metAA*, *metE* and *cysM*) and Fe-S cluster biosynthesis (*nifU*). Downregulation was observed for genes involved in energy metabolism (*mfrA*, *mfrB* and *mfrE*) and heat shock stress responses (*grpE*, *hrcA* and *clpB*). In 24 h biofilms, upregulation was observed for genes involved in aa transport (*Cj0025*c), aa metabolism (*leuB* and *leuC*), Fe-S cluster biosynthesis (*iscS*) and oxidative stress response (*herA* and *katA*). Downregulation was observed for genes involved in energy metabolism (*mfrA*, *mfrB* and *mfrE*), including C4-dicarboxylate transporter (*dcuB*). In 48 h biofilms, upregulation was observed for genes involved in cell motility (*fliK*, *flgB*, *flgE2* and *flgG2*) and virulence (*ciaC*). Downregulation was observed for several genes related to phosphate and iron transport (*pstA*, c*fbpA*, *tonB2* and *chuD*). In 72 h biofilms, upregulation was observed for genes involved in aa metabolism (*leuB* and *leuC*) and virulence *(ciaC*). Downregulation was observed in genes involved in phosphate transport (*pstC* and *pstS*) and lipooligosaccharide (LOS) outer core biosynthesis (*Cj1136*, *neuC1* and *Cj1144c*). The distributions of DEGs and the ten most upregulated and downregulated genes for all time points are displayed in Fig. S1.

### Co-expression of genes

**Fig. 2.**
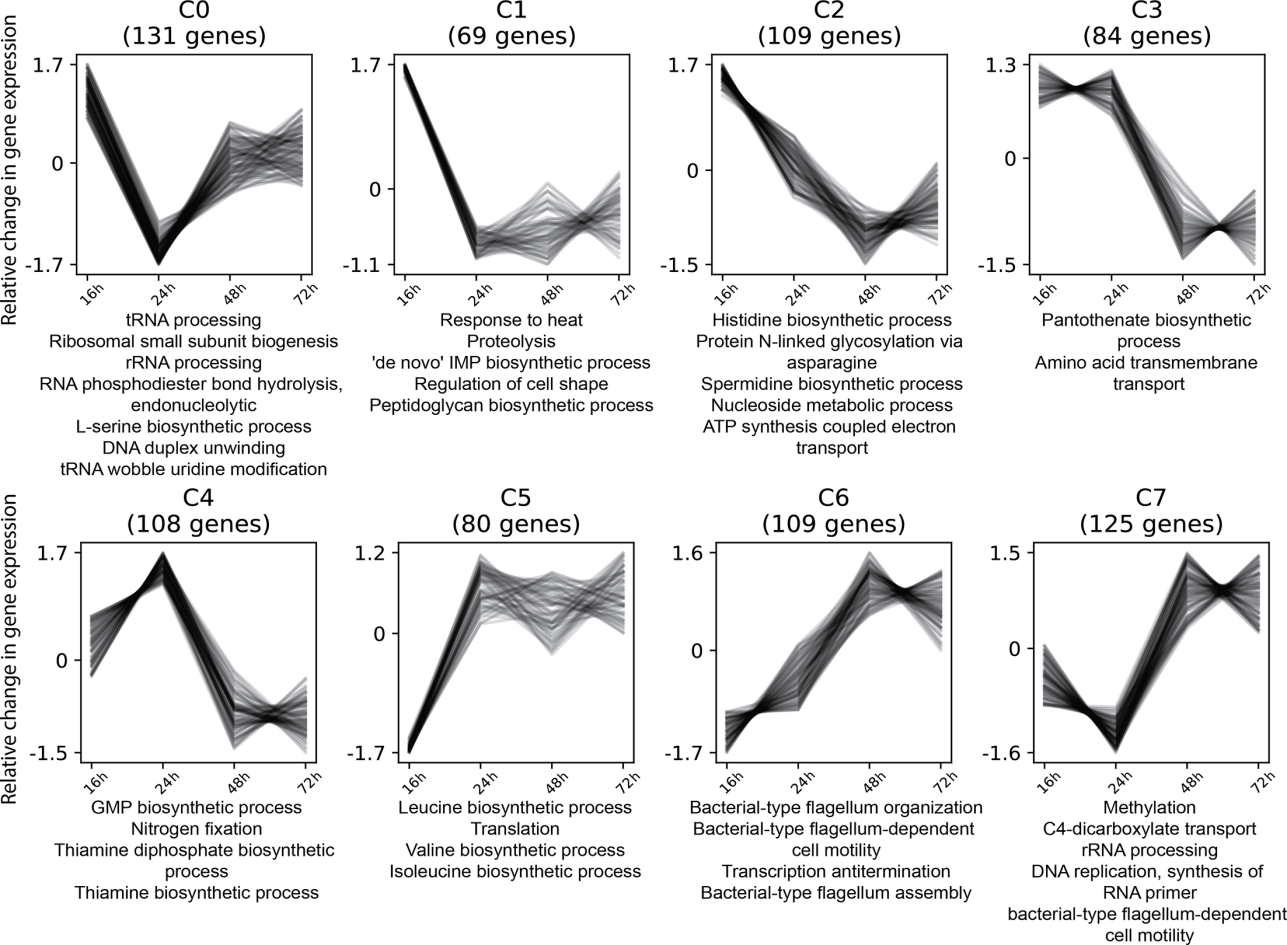
Cluster analysis of co-expressed genes and their most significant GO biological process terms. Eight co-expression clusters (C0‒C7) were identified based on relative changes in gene expression during biofilm development (16, 24, 48 and 72 h). Log_2_-fold change values were normalized. The numbers of genes in each cluster are provided in parentheses. Enriched GO biological processes are listed for each cluster, highlighting key functional categories associated with specific expression profiles. All listed GO biological processes are significantly enriched with adjusted *P*-values <0.05.

To gain insight into the co-expression patterns of genes that were expressed in a similar manner at all time points, cluster analysis was performed. Genes were grouped into eight clusters and assigned to the most significant over-representative GO terms (Fig. 2). A complete list of genes grouped into clusters is provided in Data S2. Genes with a mixed gene expression profile were assigned to clusters C0 and C4. These genes are involved in RNA/DNA processing, ribosomal biogenesis, serine biosynthesis, nt metabolism, nitrogen fixation and thiamine metabolism. Genes with decreasing expression over time were assigned to clusters C1, C2 and C3. These genes are involved in heat responses, proteolysis, nt metabolism, cell envelope biogenesis, aa transport and metabolism, energy production and pantothenate metabolism. Genes with increasing expression were assigned to clusters C5, C6 and C7. These genes are involved in branched-chain aa (leucine, isoleucine and valine) biosynthesis, translation, cell motility, transcription, methylation, C-4 dicarboxylate transport, RNA processing and DNA replication.

### Functional analysis

**Fig. 3.**
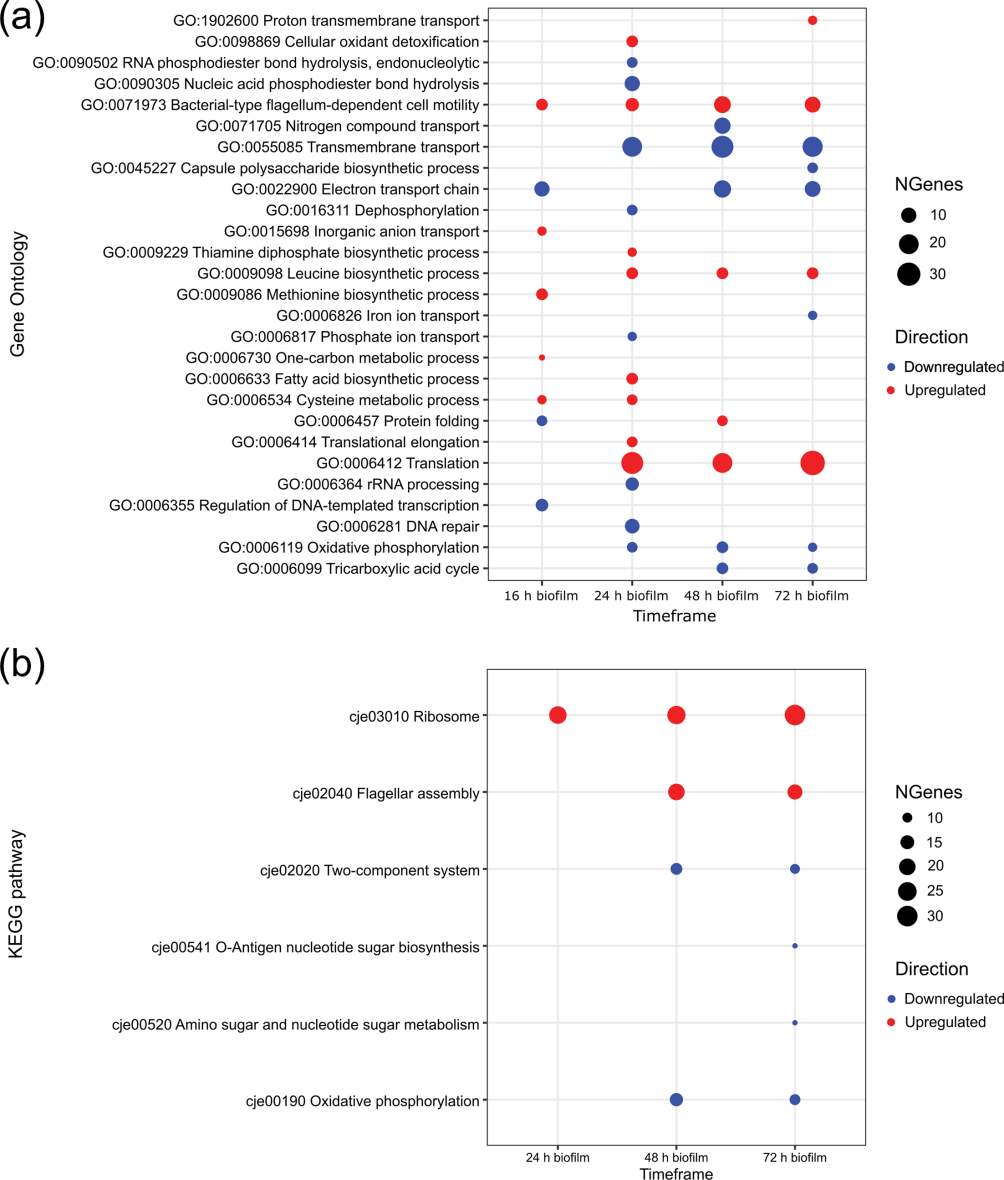
Functional enrichment analysis of DEGs with GO terms and KEGG pathways, with their corresponding ID numbers. (a) Enrichment analysis of GO biological processes. (b) Enrichment analysis of KEGG pathways. All listed categories are significantly enriched with adjusted *P*-values <0.05. Downregulated and upregulated categories are depicted in blue and red, respectively. The size of the dot represents the number of genes that were significantly expressed in each category.

To further investigate transcriptional changes during *C. jejuni* biofilm development, we conducted functional analysis by performing over-representation analysis using GO terms for biological processes and KEGG pathways (Fig. 3). In 16 h biofilms, upregulation was observed for genes involved in the aa metabolism (cysteine and methionine), one-carbon metabolism, inorganic anion transport and cell motility. Downregulation was observed for genes involved in transcription regulation, protein folding and the electron transport chain. In 24 h biofilms, upregulation was observed for genes involved in translation, cell motility, oxidative stress response and several metabolic processes, including the aa metabolism (cysteine and leucine), fatty acid biosynthesis and thiamine diphosphate biosynthesis. Downregulation was observed for genes involved in nucleic acid metabolism and processing, transmembrane transport, dephosphorylation and cellular respiration. In 48 h biofilms, upregulation was observed for genes involved in translation, protein folding, cell motility and leucine biosynthesis. Downregulation was observed for cellular respiration and transmembrane transport. In 72 h biofilms, upregulation was observed for genes involved in translation, cell motility, leucine biosynthesis and proton transmembrane transport. Downregulation was observed for genes involved in cellular respiration, iron ion transport, transmembrane transport and capsular polysaccharide (CPS) biosynthesis (Fig. 3a). The analysis of significant GO molecular function terms across all time points is provided in Fig. S2.

KEGG pathway analysis showed no enriched terms in 16 h biofilms. In 24 h biofilms, genes involved in the ribosome pathway were upregulated. In 48 h biofilms, genes involved in ribosome and cell motility pathways were upregulated, and genes involved in energy metabolism and signal transduction were downregulated. In 72 h biofilms, genes involved in ribosome and cell motility pathways were upregulated, and genes involved in energy metabolism, signal transduction, O-antigen nt sugar biosynthesis (which includes LOS genes) and amino sugar and nt sugar metabolism were downregulated (Fig. 3b).

### Transcription factor (TF) activity

**Fig. 4.**
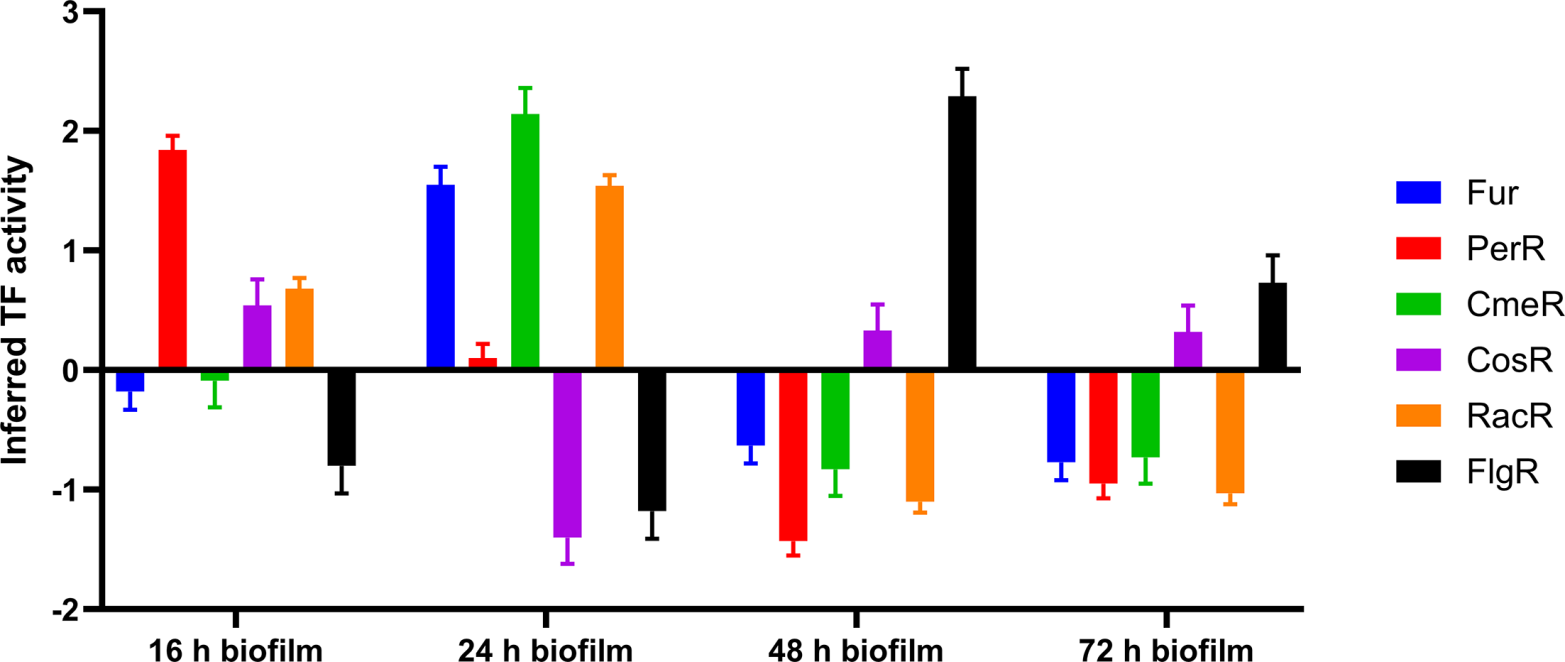
Inferred TF activity in different biofilms. The output from the TFInfer program [27] is shown for six TFs (Fur, PerR, CmeR, CosR, RacR and FlgR) across different stages of biofilm development (16, 24, 48 and 72 h). Error bars represent sd derived from the posterior distributions.

According to our analysis, significant activity was inferred for 6 out of 19 TFs: Fur, PerR, CmeR, CosR, RacR and FlgR (Fig. 4). The list of genes regulated by each TF, along with their log_2_-fold change (log_2_ FC) values, can be found in Data S2. An initial increase in TF activity (in 16 h biofilms), followed by a decrease in TF activity, was observed for PerR, which is a negative repressor of *katA* and *ahpC*, genes involved in regulating oxidative stress [17]. In 24, 48 and 72 h biofilms, *katA* and *ahpC* showed high log_2_ FC, with the highest log_2_ FC values of +3.62 and +2.76, respectively, in 24 h biofilms (Data S2). Both these genes are regulated by the Fur regulator, which showed increased activity in 24 h biofilms and decreased activity in 48 and 72 h biofilms. In 24 h biofilms, activity was decreased for the oxidative stress regulator, CosR, which negatively regulates *sodB* [18], and had a log_2_ FC of 2.81.

The highest TF activity was observed in 24 h biofilms, in which five TFs (Fur, CmeR, CosR, RacR and FlgR) had a high response (absolute activity >1; Fig. 4). The strongest increase in TF activity was observed for CmeR, a pleiotropic regulator involved in regulating the *cme* operon and many other genes with different functions [19]. The activity of CmeR decreased in 48 and 72 h biofilms. In 24 h biofilms, among the genes regulated by CmeR, the highest log_2_ FC (+3.69) was observed for *Cj0241c*, which encodes bacteriohaemerythrin, and the lowest log_2_ FC (−4.09) was observed for *mfrA*, involved in C4-dicarboxylate transport and conversion. Other genes involved in C4-dicarboxylate transport and conversion were also downregulated: *dcuA* (log_2_ FC=−2.42), *dcuB* (log_2_ FC=−3.90), *aspA* (log_2_ FC=−2.66) and *mfrA* (log_2_ FC=−4.09). These genes are involved in energy metabolism and are negatively regulated by RacR [20,21], which showed increased activity in 24 h biofilms and decreased activity in 48 and 72 h biofilms.

The strongest response was observed for FlgR in 48 h biofilms, in which the largest number of genes involved in flagellar formation was strongly upregulated. The highest log_2_ FC (+4.38) was observed for the gene *Cj0040*, which is annotated as a hypothetical protein. An InterPro scan revealed that this protein is homologous to a phosphotransferase/anion transport protein superfamily. This gene is located at the beginning of the locus for the flagellar hook protein.

## Methods

### Bacterial strains and growth conditions

*C. jejuni* NCTC11168 was stored in a solution (800 : 200 µl) of Mueller–Hinton (MH) broth (Oxoid, UK) and glycerol (Kemika, Croatia) at −80 °C. The frozen stock was transferred with a sterile loop onto Karmali agar (Oxoid, UK) supplemented with *Campylobacter-*selective Karmali supplement (Oxoid, UK). Plates were placed on a damp cloth in an airtight container (to prevent agar desiccation) and incubated in a microaerobic gas mixture (3% O_2_, 10% CO_2_ and 87% N_2_) at 42 °C. After 24 h, bacterial cultures were transferred to MH agar (Oxoid, UK) with a sterile swab and incubated for 24 h under microaerobic conditions at 42 °C. Bacteria were resuspended in MH broth to an OD at 600 nm of 0.1. For planktonic cells, 350 µl of resuspended bacteria was transferred to 7 ml of MH broth and incubated for 16 h (late exponential phase) under microaerobic conditions at 42 °C. After 16 h, cells were transferred to a 50 ml Falcon tube, mixed with a fixative consisting of 96% v/v absolute ethanol and 4% v/v phenol solution saturated with 0.1 M citrate buffer, pH 4.3±0.2 (all from Sigma-Aldrich), at a ratio of 1 : 5 (fixative:bacterial suspension), and placed on ice. For biofilm cells, 100 µl of resuspended bacteria was pipetted onto MH agar and spread out. Plates were placed in an airtight container with a damp cloth and incubated in a microaerobic gas mixture at 42 °C for 16, 24, 48 and 72 h. After incubation, 1 ml of fixative mix (1 : 5 fixative:MH broth) was pipetted onto each plate, and cells were scraped using a cell scraper. The bacterial suspension was transferred to a 15 ml Falcon tube and placed on ice. Cells were centrifuged at 5,000 ***g*** for 5 min at 4 °C, and the supernatant was removed. All experiments were conducted with four biological replicates.

### RNA isolation and quantification

RNA was isolated using a modified cetyltrimethylammonium bromide (CTAB) isolation protocol derived from a combination of two protocols [22,23] and purified using the Direct-zol RNA MiniPrep Kit (Zymo Research, USA). The RNA extraction buffer consisted of 100 mM TRIS-HCl (Promega, USA), 2 M sodium chloride (NaCl) (Merck, USA), DEPC-treated water, 25 mM EDTA disodium salt (2Na), pH 8.0, 2.5% (w/v) PVP10 MW 10,000, 2.0% (w/v) CTAB and 2.0% 2-mercaptoethanol (all from Sigma-Aldrich, USA) (added prior to RNA isolation). The phenol:chloroform:isoamyl alcohol solution was prepared with 25 ml of phenol solution, saturated with 0.1 M citrate buffer, pH 4.3±0.2, 24 ml of chloroform (Merck, USA) and 1 ml of isoamyl alcohol (Sigma-Aldrich, USA). CTAB lysis buffer (800 µl) was added to wet cell biomass (~850 mg) and vortexed for 15 s. The samples were incubated for 5 min at room temperature. After incubation, 800 µl of phenol:chloroform:isoamyl alcohol (25 : 24 : 1) solution was added to the samples and vortexed. Samples were centrifuged at 15,890 ***g*** for 5 min at 4 °C. The upper aqueous phase was carefully collected and transferred to an RNase-free microfuge tube and mixed with absolute ethanol at a ratio of 1 : 1. On-column RNA purification was conducted according to the manufacturer’s instructions, and RNA was eluted in 100 µl of RNase-free water. The RNA yield was 161–982 ng µl^−1^, and the samples were diluted to ~130 ng µl^−1^. The TURBO DNA-free Kit (Invitrogen, USA) was used to remove genomic DNA contamination using the manufacturer’s rigorous protocol. The absence of genomic DNA contamination was confirmed by agarose gel electrophoresis and qualitative PCR. Total RNA was quantified by the Qubit RNA broad-range assay (Invitrogen, USA) on a Qubit v4 fluorometer (Invitrogen, USA). RNA purity was assessed spectrophotometrically using a Lambda spectrophotometer (PerkinElmer, USA). RNA samples were considered pure if their absorption ratios were as follows: A_260_/A_280_=1.9–2.0 and A_260_/A_230_>2.0. RNA integrity was assessed with the Agilent 2100 Bioanalyzer and Agilent RNA 6000 Nano Kit (Agilent Technologies, USA).

### RNA-seq and data analysis

The preparation of a strand-speciﬁc transcriptome library and sequencing on the Illumina NovaSeq platform with 150 bp paired-end reads were conducted by Novogene (China). Raw reads containing the following were removed: (i) adapters, (ii) undetermined bases (N)>10 % and (iii) low-quality bases (Phred quality score ≤5). Read quality metrics were assessed using FastQC v0.11.9 [24]. Reads were aligned to the *C. jejuni* subsp. *jejuni* NCTC11168 (ATCC 700819) reference genome (NCBI Reference Sequence: NC_002163.1) and counted using STAR v2.7.10b [25] with default parameters. Differential gene expression was analysed in R v4.3.0 using the edgeR v3.42.4 and limma v3.56.2 packages. Raw counts were normalized using the trimmed mean of the M-value method, converted to log_2_ c.p.m. and processed using limma voom [26]. PCA of all samples (four biological replicates for five conditions) based on log_2_ c.p.m. was performed using the R built-in function prcomp. Hierarchical clustering of all samples was achieved using the hclust function with the ‘Pearson correlation method’. Biofilm samples from each time point (16, 24, 48 and 72 h) were compared against planktonic samples at 16 h. Noncoding sequences (tRNAs and rRNAs) were removed from downstream analyses. Genes with Benjamini–Hochberg false discovery rate-adjusted *P*-values of <0.05 and |log_2_ FC|≥1 were considered significantly differentially expressed. Inference of TF activities from the RNA-seq data was performed using TFInfer v1.0 [27] with a connectivity matrix of 19 TFs and 448 genes from a previous study [28].

### Functional and co-expression analyses

For functional enrichment analysis, a gene set collection containing GO or KEGG ID was created. GO data were obtained from BioCyc (https://biocyc.org/; accessed on 3 August 2023) and UniProt (https://www.uniprot.org/; accessed on 10 July 2022) and merged. Functional analysis was conducted using the BioConductor TopGO package in R (v2.52.0), which employs an over-representation analysis, using the Fisher exact test and default weight01 algorithm. Categories with weight *P*-values of <0.05 were considered significant. Over-representation analysis of KEGG pathways was conducted using the clusterProfiler v4.8.3 [29] enrichKEGG function. Categories with adjusted *P*-values of <0.05 and q-values of <0.2 were considered significant. For the identification of co-expressed gene clusters, the program clust v1.18.1 [30] was used with selected log_2_ FC normalization and a tightness parameter of 5. For the determination of genes that were over-represented in the clusters, over-representation analysis was conducted with the TopGO package. Categories with weight *P*-values of <0.05 were considered significant.

### Data visualization

The R package ggplot2 v3.5.1 was used (https://ggplot2.tidyverse.org) to create volcano and gene set enrichment bubble plots and to visualize PCA. The packages ggvenn v0.1.10 (https://github.com/yanlinlin82/ggvenn) and gplots v3.1.3 (https://github.com/talgalili/gplots) were used to create Venn diagrams and heatmaps, respectively.

## Discussion

The basis for our study was provided by two previous studies that elucidated the timelines of biofilm formation, maturation and spatial organization [12,13]. Although the timeline of biofilm development is well established, our understanding of the molecular mechanisms involved in biofilm development remains unclear. This could be addressed by determining transcriptomic variations at different stages of biofilm development [16]. Here, we report the first study to assess gene expression at different stages of *C. jejuni* biofilm development, confirming certain known key mechanisms and revealing additional new putative mechanisms.

We defined the different stages of our biofilm model based on the PCA results, which revealed five distinct transcriptomic profiles. A clear distinction can be observed between 16 h planktonic cultures, 16 h biofilms, 24 h biofilms, 48 h biofilms and 72 h biofilms, of which the latter two are more clustered together. Although 16 and 24 h biofilms exhibited different transcriptomic profiles, we will collectively refer to them as ‘early biofilms’ in our discussion, as they share most of the genes we will focus on. Similarly, we will refer to 48 and 72 h biofilms as ‘mature biofilms’.

In early biofilms, we observed a shift in aa metabolism, evident by the upregulation of genes involved in cysteine and methionine metabolism and transport (*Cj0025c*, *cjaA*, *cysM*, *metAA*, *metE*, *metF* and *metB*) and leucine metabolism (*leuABCD*). Cysteine can be synthesized *de novo* in the presence of exogenous hydrogen sulphide and thiosulphates, and methionine can be converted from cysteine. Cysteine is thought to be crucial for *C. jejuni* growth as it is required for the synthesis of proteins involved in Fe-S cluster biosynthesis [31]. Fe-S cluster complexes are present at the active sites of several key enzymes that play a crucial role in *C. jejuni* metabolism [32]. Two co-transcribed genes that are involved in the biosynthesis of Fe-S clusters in *C. jejuni* are *nifU* (encoding a scaffolding protein) and *iscS* (encoding a cysteine desulfurase) [33]. These genes showed consistent upregulation across all time points, with the highest log_2_ FC values observed in 24 h biofilms (+3.57 and +3.91, respectively). Both early and mature biofilms exhibited upregulated *leuABCD*, involved in leucine biosynthesis. Moreover, upregulated leucine metabolism was also detected in *C. jejuni* cells in the stationary phase [34]. Transcriptomic profiles of other bacteria suggest that biofilms share similar expression profiles with cells in the stationary phase [35,36]. *C. jejuni* is capable of both synthesizing leucine *de novo* and importing it via the LIV transport system [37]. Our data indicate that the expression of the LIV transporter was mostly unchanged, with some cases of downregulation, suggesting that active leucine uptake does not occur under these conditions. Since *C. jejuni* does not catabolize leucine as a carbon source [32] but uses it exclusively for protein synthesis, our results suggest that under biofilm conditions, *C. jejuni* primarily depends on endogenous leucine biosynthesis to meet its proteomic requirements.

Genes involved in the transport and utilization of C-4 dicarboxylates (*dcuAB*, *aspA* and *mfrABE*) were downregulated in early biofilms, which has already been observed in *C. jejuni* biofilms [15] and stationary-phase cells [34]. *C. jejuni* possesses a highly branched electron transport chain that enables it to utilize a variety of electron donors [38], allowing metabolic flexibility and adaptation to changing environments. The downregulation of fumarate transport and conversion is likely attributed to the lower efficiency of fumarate as an electron acceptor when other favourable electron acceptors are available [20]. Fumarate, along with pyruvate, oxaloacetate and 2-oxoglutarate, is an intermediate that is directly fed into the citric acid cycle, which is important for energy production [32]. Interestingly, the addition of fumarate to growth media promotes planktonic growth and inhibits biofilm formation of strain 81–176 [39]. According to the TF activity analysis by TFInfer, changes in the expressions of genes involved in the transport and utilization of C-4 dicarboxylates are driven by increased activities of the transcriptional regulators RacR and CmeR.

Genes involved in oxidative stress responses (*katA*, *sodA*, *ahpC*, *tpx* and *trxB*) were upregulated in early biofilms. These genes detoxify reactive oxygen species (ROS), such as superoxide anion (O_2_^-^) and hydrogen peroxide (H_2_O_2_) [40], which are not only byproducts of aerobic respiration but are also constantly produced endogenously through the autoxidation of O_2_ on a range of both aerobic and non-aerobic respiratory flavoproteins. ROS can cause damage to various cellular components, including DNA, proteins and lipids [41]. The genes *katA*, *sodA* and *ahpC* are regulated by a complex interplay of PerR, CosR, RacR and Fur [17,18, 20, 42]. According to TFInfer, PerR was not active in 24 h biofilms. The high *katA* and *ahpC* expression in this stage can be attributed to the increased activity of Fur, which regulates these two genes [17] and showed increased activity in 24 h biofilms, as revealed by TFInfer. High *sodB* expression correlates with decreased CosR activity [18]. The genes *katA* and *ahpC* remained upregulated in mature biofilms. Upregulation of genes involved in oxidative stress responses has been previously observed in *C. jejuni* [14,34], *Helicobacter pylori* [43] and *Pseudomonas aeruginosa* [44].

Interestingly, *C. jejuni* upregulates these oxidative stress genes not only under oxygen-rich or microaerobic biofilm conditions [45] but also in response to various other environmental stresses, such as temperature, acid, osmotic and starvation stresses [40]. The activation of *katA*, *sodB*, *ahpC* and other oxidative stress response genes in biofilms aligns with their broader role in counteracting ROS accumulation under diverse stress conditions. Biofilm growth reduces the penetration of nutrients into inner layers and limits the diffusion of metabolic wastes, resulting in nutritional limitation and physiological stress [46]. Consequently, upregulation of these oxidative stress genes likely mitigates ROS-related damage in biofilms.

In mature biofilms, especially in 72 h biofilms, genes involved in the CPS and LOS outer core were downregulated. LOS consists of short-chain sugar residues linked to the lipid A component in the outer membrane, influences bacterial interactions with the host or environment and contributes to outer membrane stability. In *C. jejuni*, the LOS biosynthesis cluster extends from *waaC* (*Cj1133*) to *cyf* (*Cj1153*) and contains genes involved in the biosynthesis of the inner and outer LOS cores, of which the outer core (*Cj1136–Cj1145c*) is a hypervariable region [5,47]. Deletions of various genes encoding the outer LOS core enhance biofilm formation in strain 81–176 [48].

CPS forms the outermost layer of many bacteria and plays an important role in the interactions between the bacterium, host and environment. In *C. jejuni*, genes encoding CPS are highly variable due to phase variation [49]. The effects of CPS gene mutations on *C. jejuni* virulence are strain- and cell-model-specific. For instance, an acapsular mutant *kpsM* (involved in CPS export) is less virulent in strain 81–176 than in strain 11168, which exhibits increased adhesion and invasion [50]. The effects of CPS gene mutations on *C. jejuni* biofilm formation also appear to be strain-specific, as deletion of *kpsM* increases biofilm production in strain 81–176 [51] and decreases biofilm production in strain 11168, which was, however, only investigated at later time points [50]. The CPS biosynthesis cluster in strain 11168 spans from *kpsS* (*Cj1413*) to *kpsM* (*Cj1448*) [52]. CPS are generally associated with reduced adherence and biofilm formation, as CPS-deficient mutants of *Streptococcus pneumoniae*, *Neisseria meningitidis*, *Staphylococcus aureus* and *Vibrio vulnificans* exhibit enhanced attachment to epithelial cells and increased biofilm development. CPS expression is often downregulated upon contact with epithelial surfaces and during biofilm formation [53]. Similarly, *C. jejuni* downregulates CPS when cocultured with epithelial cells [54], suggesting a host-adaptive response. Our data indicate that approximately half of this cluster is strongly downregulated in mature biofilms, supporting the role of CPS modulation beyond host adaptation and its involvement in biofilm development.

Loss of CPS and truncation of LOS increase surface hydrophobicity and DNA uptake, possibly because LOS can act as an electrostatic barrier (both polysaccharides and DNA are negatively charged) or a physical barrier, hindering the binding of DNA to receptors on the outer membrane [55]. Increased DNA uptake was previously observed in *C. jejuni* biofilms, with eDNA being the potential DNA substrate [13]. Interestingly, the genes *comCE* (*Cj1211*), involved in natural transformation, and *Cj0683*, implicated in the competence (pseudo)-pilus [56], were upregulated in our mature biofilms. This suggests that *C. jejuni* may regulate LOS/CPS in the later stages of biofilm development to accommodate the increased demand for DNA uptake/transformation. As LOS/CPS can provide a physical barrier, downregulation of these genes may also facilitate nutrient uptake in *C. jejuni* biofilms.

In both early and mature biofilms, flagellar genes were strongly upregulated. According to TFInfer, FlgR activity was observed in mature biofilms, when the number of flagellar genes increased compared to that of early biofilms. In addition to FlgR (part of the two-component system FlgR/FlgS), σ^28^ factor (*fliA*), σ^54^ factor (*rpoN*), anti-σ factor FlgM and FlhF GTPase [57] also regulate flagellar genes, possibly influencing the upregulation of flagellar genes in early biofilms. The upregulation of flagellar genes that we observed is consistent with the literature, as continuous expression of these genes has been observed in *C. jejuni* during biofilm formation [14] and even in stationary-phase cultures [34]. For adhesion and biofilm formation, a flagellar structure is required; however, its functionality is not crucial [13]. Flagella may also play a structural role in *C. jejuni* biofilms by acting as bridges in net-like connections between cells [58], and such a role was proposed during the biofilm formation of *H. pylori* (a close relative of *C. jejuni*) [43]. Similar structural functions have been observed in *Escherichia coli* macrocolony biofilms, where flagella tether cells together in the lower biofilm layers and contribute to the mechanical integrity of the biofilm. Their entanglement, driven by rotation, further stabilizes bacterial communities within macrocolony biofilms [59]. In *E. coli* pellicle biofilms, flagella have also been detected within the extracellular matrix, where they likely assist in biofilm stabilization by interacting with other matrix components [60]. These findings suggest that beyond adhesion, flagella act as key structural elements in *C. jejuni* biofilms, enhancing biofilm architecture and mechanical stability.

The observed downregulation of genes involved in the electron transport chain and oxidative phosphorylation suggests altered energy metabolism in *C. jejuni* biofilms. In mature biofilms, genes involved in the citric acid cycle were also downregulated. To fulfil its energy requirements, *C. jejuni* relies on the citric acid cycle and can directly utilize its various intermediates as nutrient sources [32]. Downregulation of the citric acid cycle at later time points may indicate changes in nutrient availability of the catabolizable substrates within the biofilm microenvironment and/or lower energy requirements of cells, as oxidative phosphorylation was also downregulated. Moreover, this downregulation may serve as a strategy to reduce ROS production, which, as previously discussed, is an unavoidable consequence of respiration when O_₂_ is present [41]. Downregulation of genes involved in electron transport and the citric acid cycle was also observed in *H. pylori* biofilms [61], supporting the concept that microaerophilic pathogens may adopt a lower-energy metabolic state during biofilm maturation.

Downregulated transmembrane transport was common in both early and mature biofilms. Genes included in this functional category encode several transporters, including the efflux system CmeDEF (*Cj1031–Cj1033*), phosphate transport complex PstSACB (*Cj0613–Cj0616*) and iron transport complexes. Transcription of the *pstSACB* complex is activated under phosphate-limiting conditions [62]. However, this does not appear to be the case in our biofilm model, as we used complex media in our experiments. *C. jejuni* has evolved several iron acquisition pathways [32], of which many were downregulated in our biofilms, including systems for ferric-enterobactin (*ceuBCDE*/*Cj1352–Cj1355*), haem (*ChuABCD*/*Cj1614–Cj1617*), ferric-rhodotorulic acid (p19/*Cj1659*, *Cj1658–Cj1663*), ferric-transferrin/lactoferrin (CfbpABC/*Cj0173c–Cj1735c*) and ferrous ion transporter *feoB* (*Cj1398*). The downregulation of iron acquisition systems in our biofilm models suggests iron-repleted conditions, as these genes are upregulated under iron-limited conditions. Regulation of iron homeostasis is crucial as excess iron triggers oxidative stress via the Fenton reaction [63]. Based on the inferred TF activity of Fur, we could not explain the downregulation of iron transport genes, as most iron-responsive genes in *C. jejuni* are not directly or indirectly regulated by Fur [63,64].

**Fig. 5.**
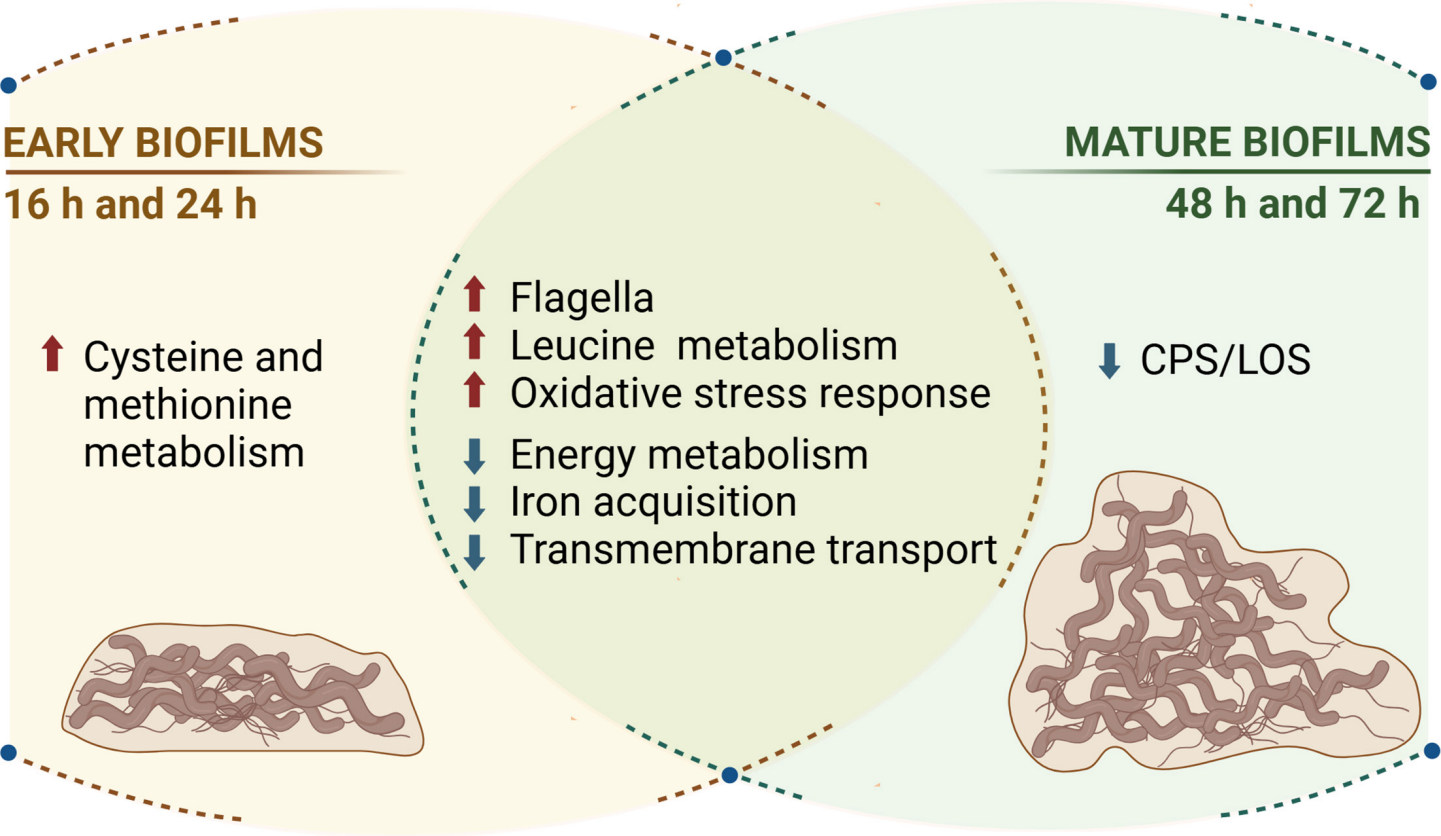
Graphical summary of gene expression profiles in early and mature biofilms of *C. jejuni*. The figure shows the upregulation (red arrows) and downregulation (blue arrows) of key functional categories at different biofilm stages, with early biofilms at 16 and 24 h and mature biofilms at 48 and 72 h.

Taken together, early and mature biofilms are characterized by increased expression of genes involved in flagella, leucine aa metabolism and oxidative stress responses and decreased expression of genes involved in energy metabolism, iron acquisition and transmembrane transport. Furthermore, early biofilms show increased expression of genes involved in cysteine and methionine aa metabolism, whereas mature biofilms show decreased CPS/LOS expression. A graphical summary of these findings is depicted in Fig. 5. These distinct expression profiles of different biofilm stages underline the importance of temporally analysing gene expression, as this provides a more detailed understanding of the processes involved in biofilm formation and maturation.

Biofilm formation in *C. jejuni* is influenced by a complex interplay of environmental factors, including oxygen levels, nutrient availability and multiple species interactions, as well as transcriptional regulation, phase variation and strain-specific genetic differences [65,66]. The observed upregulation of oxidative stress genes and downregulation of CPS at later time points suggest that *C. jejuni* dynamically adjusts its surface structures and stress responses in biofilms, likely as an adaptation to nutrient limitation, metabolic waste accumulation and other microenvironmental pressures.

Biofilm formation in *C. jejuni* contributes to environmental persistence by protecting cells from oxidative stress, nutrient limitation and other external pressures [11,65, 67]. However, its direct role in *in vivo* virulence remains less defined. One study reported that biofilm-derived *C. jejuni* exhibited reduced colonization efficiency in young chickens compared to planktonic cells, suggesting that biofilm-associated phenotypes do not necessarily enhance pathogenicity. In contrast, *in vitro* virulence assays in cell culture models yielded inconsistent results, with variations depending on the strain and cell model used in the study [68]. Moreover, there is no clear evidence that *C. jejuni* forms biofilms within the gut or other host tissues, making it difficult to directly link biofilm formation to infection dynamics [4]. Nevertheless, biofilms enhance *C. jejuni* survival in external environments, such as food-processing facilities and water systems, which may indirectly increase transmission risk. Biofilm cells can persist on abiotic surfaces or in water for prolonged periods, facilitating the recontamination of livestock, including poultry, or direct infection through contaminated food or water [65].

Our biofilm models consist of biofilms on agar plates grown under optimal conditions. However, biofilm formation varies among *C. jejuni* strains, with host generalists (strains capable of colonizing multiple host species) often exhibiting stronger biofilm phenotypes than host specialists [66]. Biofilms are particularly relevant for strains cycling through multiple hosts or persisting in food-processing environments and water systems, where they facilitate survival and transmission [65]. Given the variability in biofilm formation among *C. jejuni* strains and their role in environmental persistence, future studies should investigate the dynamics of biofilm formation on different abiotic surfaces under both conditions that promote biofilm formation and those that simulate real food-processing environments. Comparative studies across diverse *C. jejuni* lineages could further elucidate how strain-specific differences influence biofilm formation, persistence and transmission. Such research will contribute to a more comprehensive understanding of the molecular mechanisms and adaptive strategies of *C. jejuni* in diverse environments.

## Supplementary material

10.1099/mgen.0.001387 Uncited Supplementary Material 1.

10.1099/mgen.0.001387 Uncited Supplementary Data Sheet 1.

10.1099/mgen.0.001387 Uncited Supplementary Data Sheet 2.

## Abbreviations

CPS: capsular polysaccharide
CTAB: cetyltrimethylammonium bromide
DEGs: differentially expressed genes
eDNA: environmental DNA
EDTA: ethylenediaminetetraacetic acid
Fe-S: iron–sulfur
GO: Gene Ontology
KEGG: Kyoto Encyclopedia of Genes and Genomes
log_2_ FC: log_2_-fold change
LOS: lipooligosaccharide
MH: Mueller–Hinton
NaCl: sodium chloride
OD600: optical density at 600 nm
PCA: principal component analysis
RNA-seq: RNA sequencing
ROS: reactive oxygen species
TF: transcription factor
