# Supplementary material for: Temporal dynamics of gene expression during the development of Campylobacter jejuni biofilms
Source: Microb Genom. 2025 May 6;11(5):001387. doi: 10.1099/mgen.0.001387 (PMC12056249; doi:10.1099/mgen.0.001387)
Supplement: Uncited Supplementary Material 1. [file mgen-11-01387-s001.pdf]

Table S1. **Aligned and mapped reads.** The percentage of aligned reads and the number of uniquely mapped reads (in millions) for RNA samples extracted from 16 h planktonic cultures and 16 h, 24 h, 48 h, and 72 h biofilms.

Sample Name	% Aligned	Uniquely mapped reads (in million)
16 h_1	87.2%	6.6
16 h_2	88.5%	6.7
16 h_3	89.2%	7.8
16 h_4	89.4%	8.2
16 h biofilm_1	88.0%	7.3
16 h biofilm_2	85.1%	5.7
16 h biofilm_3	90.1%	7.8
16 h biofilm_4	90.8%	9.9
24 h biofilm_1	91.3%	9.2
24 h biofilm_2	37.2%	3.8
24 h biofilm_3	81.3%	8.2
24 h biofilm_4	90.7%	6.2
48 h biofilm_1	85.1%	7.2
48 h biofilm_2	85.9%	7.4
48 h biofilm_3	82.0%	7.7
48 h biofilm_4	76.5%	5.7
72 h biofilm_1	70.4%	4.9
72 h biofilm_2	81.7%	7.6
72 h biofilm_3	83.5%	5.9
72 h biofilm_4	82.4%	5.6

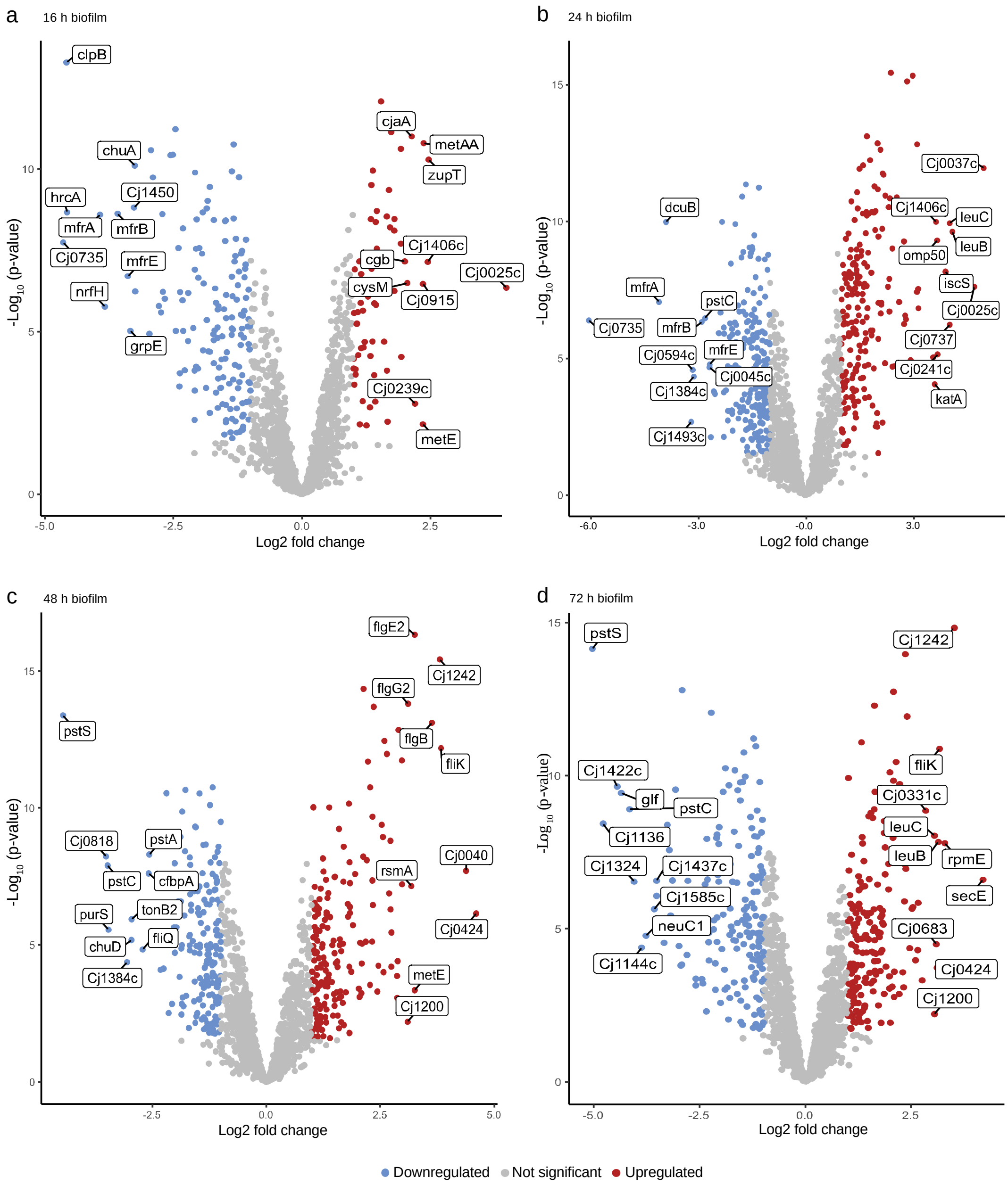

Fig. S1. Volcano plots of differentially expressed genes in biofilms at four time points compared to the planktonic culture. a) 16 h biofilms, b) 24 h biofilms, c) 48 h biofilms, and d) 72 h biofilms. Upregulated genes (log_2-fold change > 1 and adjusted p-value < 0.05) and downregulated genes (log_2-fold change < −1 and adjusted p-value < 0.05) are indicated in red and blue, respectively. Non-significant genes are shown in grey. The top 10 differentially expressed genes are labelled in each plot.

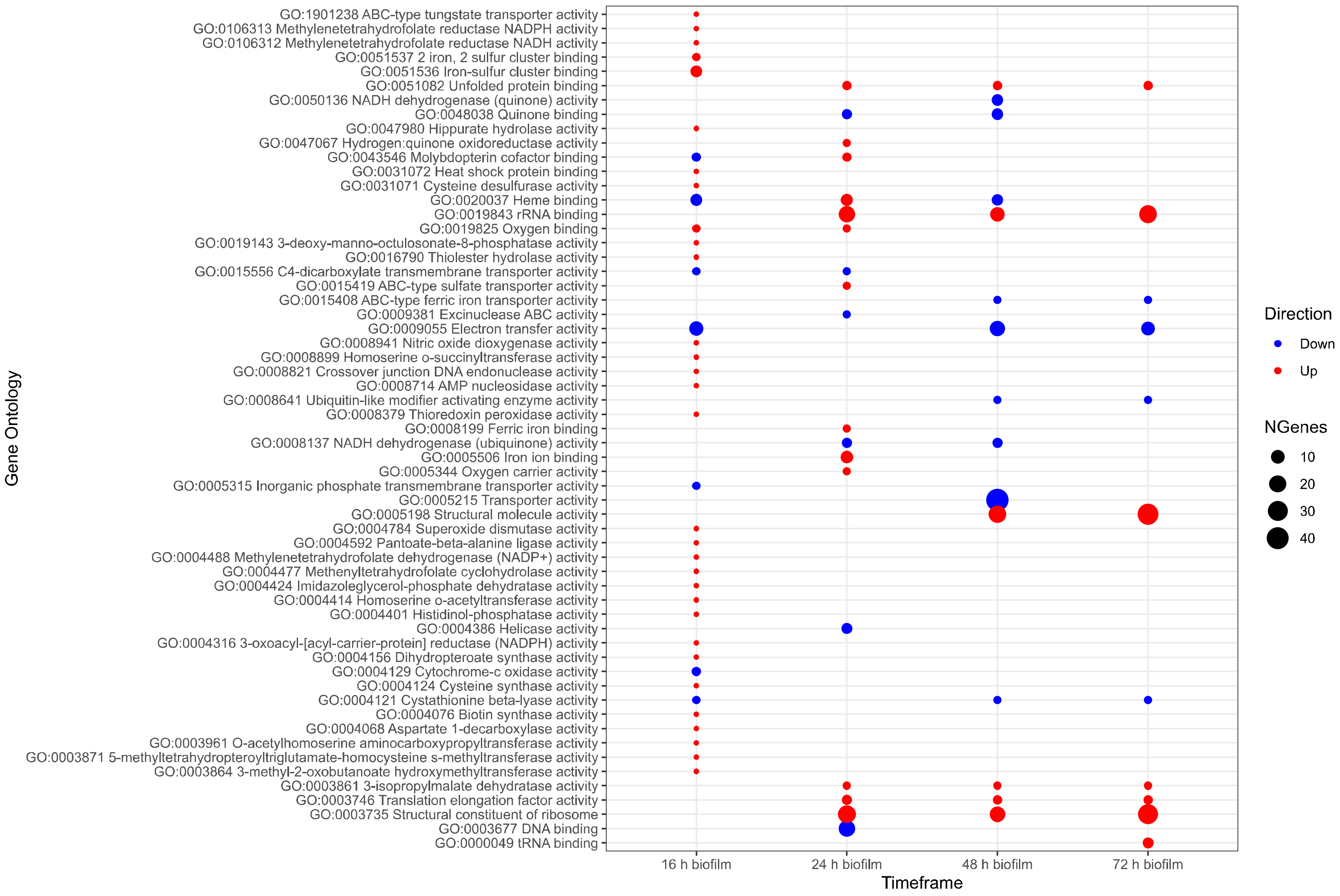

Fig. S2. Functional enrichment analysis of differentially expressed genes with gene ontology (GO) molecular function terms. All listed categories are significantly enriched with adjusted p-values < 0.05. Downregulated and upregulated genes are depicted in blue and red, respectively. The size of the dot represents the number of genes that were significantly expressed in each category.
